# Editorial: Neuroprotection and disease modification in Parkinson’s disease: Volume II

**DOI:** 10.3389/fphar.2022.1121513

**Published:** 2023-01-09

**Authors:** Matilde Otero-Losada, Paolo Gubellini, Francisco Capani, Santiago Perez-Lloret

**Affiliations:** ^1^ Consejo Nacional de Investigaciones Cientificas y Tecnicas, Universidad Abierta Interamericana-Centro de Altos Estudios en Ciencias Humanas y de La Salud, UAI-CAECIHS CONICET, Buenos Aires, Argentina; ^2^ CNRS, IBDM, UMR7288, Aix-Marseille University, Marseille, France; ^3^ Instituto de Ciencias Biomedicas, Facultad de Ciencias de la Salud, Universidad Autonoma de Chile, Santiago de Chile, Chile; ^4^ Observatorio de Salud Pública, Pontificia Universidad Católica Argentina (UCA), Buenos Aires, Argentina; ^5^ Department of Physiology, Faculty of Medicine, University of Buenos Aires (UBA), Buenos Aires, Argentina; ^6^ Consejo Nacional de Investigaciones Científicas y Técnicas (CONICET), Buenos Aires, Argentina

**Keywords:** neuroprotection, disease-modifying therapeutics, pathophysiology, natural history, Parkinson’s disease

Parkinson’s disease (PD) is the second prevailing neurodegenerative disease worldwide after Alzheimer’s. Nearly 1% of seniors over 65 suffer from this disease, and as old population grows rapidly, so does PD incidence. Early onset PD, before the 50s, is usually inherited, likely related to specific gene mutations. Added to the characteristic symptoms of PD, v.g., bradykinesia, shaking, stiffness, and handicapped balance and coordination, innumerable non-motor symptoms like changes in mood, sleep patterns and quality, and autonomic alterations impair patients’ life quality. Current treatments aim to mitigate PD symptoms, not decelerating disease progression. So far, no preventing approach is available against PD.

Current pharmacology in this field has a clear priority, which is to identify or develop drugs and therapies to hold PD progression. Basic research to test the potential of new molecules and strategies in neuroprotection is thus mandatory.

This Research Topic addresses some new pharmacological neuroprotective and disease-modifying strategies for PD, including possible target pathways, and effects on humans. We trust this updated information may help to devising new therapeutic strategies to prevent or delay PD onset and progression.

Subjects covered in this Research Topic are:

*Peripheral Neuroprotective and Immunomodulatory Effects of 5α-Reductase Inhibitors in Parkinson’s Disease Models*. After finding an immunomodulatory effect of female hormones to treat enteric neurodegeneration in the 1-methyl-4-phenyl-1,2,3,6-tetrahydropyridine (MPTP) mouse model of PD, Poirier et al. tested the hypothesis of obtaining similar neuroprotection by affecting steroidogenesis with two 5α-reductase inhibitors, finasteride and dutasteride. They found that dutasteride treatment prevented enteric neuronal damage in the MPTP mouse model, partly *via* anti-inflammatory and mitochondrial effects. Dutasteride might thus constitute a promising drug for treating enteric neuroinflammation in early PD (Perez-Pardo et al., 2019; Poirier et al., 2016).

*Characterization of Nasco grape pomace-loaded nutriosomes and their neuroprotective effects in the MPTP mouse model of Parkinson’s disease*. The neuroprotective effects of Nasco pomace, rich in the antioxidant polyphenols gallic acid, catechin, epicatechin, procyanidin B2, and quercetin, were investigated in the subacute MPTP mouse model of PD. Nasco nutriosomes or Nasco suspension was administered intragastrically. The results proved neuroprotection, validating the effectiveness of the nutriosome preparation over suspension as an innovative nano-drug delivery system for *in vivo* administration (Parekh et al.) (Pazos-Tomas et al., 2020; Shahpiri et al., 2016).

*Preclinical reserpine models recapitulating motor and non-motor features of Parkinson’s disease: Roles of epigenetic upregulation of alpha-synuclein and autophagy impairment*. Li et al. used a preclinical animal model using reserpine to recapitulate motor and non-motor PD symptoms. Chronic reserpine exposure caused hypomethylation of the alpha-synuclein gene, upregulated its expression, and elevated the LC3-II/LC3-I ratio and expression of p62 in mice substantia nigra. Chronic reserpine exposure epigenetically raised alpha-synuclein expression, likely *via* decreasing DNA methylation and inducing autophagic impairment *in vitro* and *in vivo* (Chia et al., 2020; Fernandes et al., 2012).

*Motor cortico-nigral and cortico-entopeduncular information transmission and its modulation by buspirone in control and after dopaminergic denervation*. The nigrostriatal dopamine (DA) and the activation of 5-HT1A receptors, distributed in the basal ganglia, may modulate cortical information transmission. The authors investigated the effect of buspirone (5-HT1A receptor partial agonist) and WAY-100635 (5-HT1A receptor antagonist) on cortico-nigral and cortico-entopeduncular transmission in normal and DA loss conditions. In control condition, buspirone potentiated direct pathway transmission and DA loss modulated responses related to the hyperdirect pathway. The results may help to clarify the role of 5-HT1A receptors and DA in motor cortico-basal ganglia circuitry functionality (Vegas-Suárez et al.) (Miguelez et al., 2014; Suarez et al., 2020).

*Epigallocatechin-3-gallate: A phytochemical as a promising drug candidate for the treatment of Parkinson’s disease* (frontiersin.org). Wang et al. compiled recent updates and knowledge of the molecular mechanisms underlying the neuroprotective effects of Epigallocatechin 3-gallate (EGCG), an abundant polyphenolic component derived from green tea extract in PD. They focused on EGCG effects on apoptosis, oxidative stress, inflammation, ferroptosis, dopamine synthesis modulation, and α-synuclein the aggregation. So far, the evidence reveals a promising role of EGCG in neuroprotection in PD treatment (Mahoney-Sánchez et al., 2021; Martinez-Perez et al., 2018).

*Adverse event profiles of adjuvant treatment with opicapone in Parkinson’s disease: A systematic review and meta-analysis*. Tolerability and treatment-related adverse events of short-term (<6 months) and long-term (≥6 months) treatment with opicapone, a novel third-generation catechol-O-methyltransferase inhibitor, were compared in PD patients. Overall, opicapone was usually well-tolerated, with a low risk of adverse events including dyskinesia (16.1%), dry mouth (12.1%), decreased medication effect (12.1%), exacerbated PD (7.8%), increased blood creatine phosphokinase level (7.4%), nausea (6.1%) and insomnia (5.1%) (Xie et al.) (Żegleń et al., 2022; Takeda et al., 2021).

*Doxycycline attenuates l-DOPA-induced dyskinesia through an anti-inflammatory effect in a hemiparkinsonian mouse model*. Targeting neuroinflammation seems promising in alleviating l-DOPA-induced dyskinesia (LID) in PD. In this study, doxycycline, a semisynthetic brain-penetrant tetracycline antibiotic with interesting anti-inflammatory properties, was found to prevent LID regardless of the severity of dopaminergic lesioning in an experimental mice model, likely due to its anti-inflammatory effects. Doxycycline poses as an attractive and alternative treatment for LID in PD (dos Santos Pereira et al.) (Wang et al., 2017; Santa-Cecilia et al., 2016).

Cutting edge research is crucial for identifying and testing new compounds and strategies for, if healing is not possible, at the least, alleviating PD nuisance. To end, a few words, recalling Hippocrates: “I will remember that there is art to medicine as well as science, and that warmth, sympathy, and understanding may outweigh the surgeon’s knife or the chemist’s drug” [Greek Medicine-The Hippocratic Oath (nih.gov)].
